# Adversarial Range Gate Pull-Off Jamming Against Tracking Radar

**DOI:** 10.3390/s25051553

**Published:** 2025-03-03

**Authors:** Yuanhang Wang, Yi Han, Yi Jiang

**Affiliations:** 1 School of Information and Communication Engineering, Dalian University of Technology, Dalian 116024, China; dlutjy@mail.dlut.edu.cn; 2 School of Information and Communication Engineering, University of Electronic Science and Technology of China, Chengdu 611731, China; 201911012002@std.uestc.edu.cn

**Keywords:** range gate pull-off (RGPO), radar tracking system, adversarial stochastic simulation optimization (ASSO), particle swarm optimization with equal resampling scheme (PSO-ER)

## Abstract

Range gate pull-off (RGPO) jamming is an effective method for track deception aimed at radar systems. Nevertheless, enhancing the effectiveness of the jamming strategy continues to pose challenges, restricting the RGPO jamming method from achieving its maximum potential. This paper focuses on addressing the problem of optimizing the strategy for white-box RGPO jamming, serving as a foundational step toward quantitative optimization research on RGPO jamming strategies. In the white-box scenario, it is presumed that the jammer has full knowledge of the target radar’s tracking system, encompassing both the choice of tracking method and its parameter configurations. The intricate interactions between the jammer and the tracking radar introduce three primary challenges: **(1)** Formulating an algebraic expression for the objective function of the jamming strategy optimization is nontrivial; **(2)** Direct observation of jamming effects from the target radar is challenging; **(3)** Noise renders the jamming outcomes unpredictable. To tackle these challenges, this study formulates the optimization of the RGPO jamming strategy as an adversarial stochastic simulation optimization (ASSO) problem and introduces a novel solution for the white-box RGPO jamming strategy optimization: a local simulation-assisted particle swarm optimization algorithm with an equal resampling scheme (PSO-ER). The PSO-ER algorithm searches for optimal jamming strategies while utilizing a localized simulation of the tracking radar to evaluate the effectiveness of candidate jamming strategies. Experiments conducted on four benchmark cases confirm that the proposed approach is capable of generating well-tuned strategies for white-box RGPO jamming.

## 1. Introduction

Radar deception remains a focal area of research within the field of electronic countermeasures (ECMs) [1,2,3,4,5,6,7]. Among various techniques, range gate pull-off (RGPO) jamming is regarded as a highly effective approach for deceiving radar tracking systems [5,6,7,8,9]. However, designing an appropriate RGPO jamming strategy is crucial for achieving successful track deception against the threat radar [3]. During each jamming stage (i.e., the tracking frame), the jammer begins by capturing the radar’s transmitted signal and then retransmitting it back to the radar receiver with a deliberately manipulated delay. The retransmitted signal (i.e., the jamming signal) will blend into the radar’s received signal, resulting in the radar detecting a false target. When the delay is carefully adjusted for each frame, the false target’s measurement enters the tracking gate, leading to errors in the radar’s tracking of the real target. Moreover, when delays across successive tracking frames are systematically coordinated, the tracking error rapidly accumulates. Consequently, the tracking gate diverges significantly from the real target’s position. Ultimately, this causes the radar to incorrectly follow the false target, losing track of the real one entirely. On the contrary, if the delays of all jamming stage (i.e., RGPO jamming strategy) are set arbitrarily, the jamming signal can only form false measurement points separated from each other among frames at the radar tracking end. In this case, it is almost impossible to deceive the threat radar to form a false track and lose the real target. Therefore, it is necessary to study the setting of the RGPO jamming strategy.

In recent years, certain scholars have conducted qualitative investigations into the optimization challenges associated with RGPO jamming strategies [3]. A frequently adopted approach involves formulating an algebraic optimization model and applying corresponding algorithms to derive the best RGPO jamming strategy. Nonetheless, the intricate interactions between the jammer and the threat radar make it challenging to formulate an algebraic representation of the objective function. In [10], we preliminarily study data-driven optimization of RGPO jamming strategies. However, the main assumptions in [10] are simple and ideal, and problems such as data acquisition and the limitations of the optimizer in actual use are not fully considered. As a result, the research on the RGPO jamming strategy optimization yields weak results. In practice, uniform velocity RGPO (UV-RGPO) and uniform acceleration RGPO (UA-RGPO) are two of the most popular ways to set the RGPO jamming strategy [6,7]. For the UV-RGPO, the delays are controlled so that the false target is separated from the real target with uniform velocity motion. Similarly, for the UA-RGPO method, the delays are controlled so that the false target is separated from the real target with uniform acceleration motion. Nonetheless, the setting of the key parameters, such as pull-off velocity for the UV-RGPO, initial pull-off velocity, and pull-off acceleration for the UA-RGPO, still relies on expert experience. Consequently, the capability of RGPO jamming as an effective track-deception technique remains significantly underutilized.

The strategy optimization of the RGPO jamming faces three main difficulties: **(1)** Because of the intricate, multi-stage confrontation between the jammer and the tracking radar, it is difficult to obtain the algebraic description of the objective function; **(2)** Due to the adversarial relationship between the jammer and the tracking radar, it is difficult for the jammer to observe jamming results directly from the threat radar; **(3)** The presence of noise, including process and measurement noise, renders the jamming outcomes indeterministic. For such complex optimization problems, stochastic simulation optimization (SSO) technology may be a powerful tool [11,12,13,14,15]. In general, algebraic descriptions of SSO problems are difficult to obtain due to the high complexity of problems, and the output of the problems is not deterministic due to the influence of noise. Thus, SSO algorithms only rely on input–output data to search for the best solution. In the real world, many complex optimization problems can be regarded as SSO problems, such as power system designing [12] and wafer probe testing [15]. As a result, SSO technology has been widely used in these fields. From the aforementioned difficulties **(1)** and **(3)**, we can find that the optimization of RGPO jamming strategies is largely applicable to be described as an SSO problem. However, different from the canonical SSO problems, it is difficult for the RGPO jamming to directly obtain input–output data to support the strategy optimization due to difficulty **(2)**. Therefore, the optimization of RGPO jamming strategies can be seen as a more complex adversarial SSO (ASSO) problem.

Compared with the canonical SSO problems, the challenge of the ASSO problems lies in the acquisition of the input–output data. A research field that may provide reference is adversarial attacks on deep neural networks (DNNs). In adversarial attacks on DNNs [16,17,18,19], the attacker fools the attacked DNN by adding an amount of artificial perturbations to the original input, which can biases the output of the attacked DNN. To obtain the desired attack effect, the attack strategy needs to be carefully designed [17]. However, due to the complexity of DNN, it is difficult to get the algebraic description of the objective function [19]. Moreover, for the attacker, it is difficult to obtain the output of the attacked DNN. To solve the above problems, the researchers propose to build a local simulation network of the attacked DNN to support the optimization of the attack strategy. Moreover, for the convenience of research, the adversarial attacks on DNNs are divided into white-box and black-box attacks, based on the amount of knowledge about the attacked DNNs [20,21,22]. In white-box attacks, the attacker has the full knowledge of the attacked DNN, while in black-box attacks the attacker does not have knowledge of the attacked DNN. For the white-box attack, a local simulation network of the attacked DNN can be built easily. For the black-box attack, the establishment of a local simulation network is a more complex issue. However, research on the white-box attack is the foundation for the research on the black-box attack in the optimization model and the main framework of the algorithm.

Comparing RGPO jamming and adversarial attacks on DNNs, it is not difficult to find that the fundamental content of them is very similar. Specifically, the jammer can be seen as the attacker, the threat radar plays the same role as the attacked DNN, the RGPO jamming signals can be seen as the artificial perturbations added to the original input, and the radar echo signal is the original input. Therefore, RGPO jamming can be analogized as an adversarial attack on the threat radar. Drawing inspiration from studies on adversarial attacks against DNNs, we classify RGPO jamming into two types: white-box and black-box jamming, depending on the jammer’s knowledge of the threat radar’s tracking model (e.g., tracking methods, parameters, etc.). The white-box scenario assumes that the jammer possesses detailed information about the tracking model of the threat radar. By contrast, the black-box scenario presumes that the jammer lacks access to precise details of the tracking model. Since the white-box scenario serves as the foundation for exploring black-box scenarios, this paper primarily focuses on addressing the optimization challenge for white-box RGPO jamming strategies. Similar to white-box attacks on DNNs, in the context of white-box RGPO jamming, a simulated tracking system of the threat radar can be constructed locally to produce input–output data, supporting the optimization of jamming strategies. To this end, we introduce a local simulation assisted SSO algorithm for solving the strategy optimization problem of the white-box RGPO jamming.

Generally, SSO algorithms are the integrations of metaheuristic algorithms (MAs) and resampling schemes [11,23]. MAs are responsible for searching the strategy space and generating candidate strategies for comparison, while resampling schemes are used to mitigate the influence of noise on the performance estimation of candidate strategies. Particle swarm optimization (PSO) is a popular MA algorithm and shows a very high search efficiency in many real-world problems [24]. Meanwhile, equal resampling (ER) represents a straightforward method to reduce the impact of noise [25,26,27]. This approach assigns an equal number of resamples to each candidate strategy (i.e., repeated evaluations of candidate strategy performance using the local simulation system). The average performance across these resamples is then used to estimate the final effectiveness of the candidate strategies. Therefore, we propose a strategy optimization algorithm for white-box RGPO jamming that integrates a local simulation model and a PSO algorithm with an ER scheme (PSO-ER).

The main contributions of this research are as follows:

1. We formulate the strategy optimization of the RGPO jamming as an ASSO problem, which lays the foundation for the nature of the problem and the optimization model for subsequent related research.
2. We propose an idea to use local simulation assisted SSO algorithms to solve the strategy optimization problem for RGPO jamming, which lays the foundation for the main frame of the algorithm for subsequent related research.
3. In this paper, an optimization algorithm based on PSO-ER with local simulation assistance is proposed to generate well-tuned strategies for white-box RGPO jamming.

The remainder of this paper is organized as follows: Section 2 elaborates the process of RGPO jamming and formulates the optimization problem. Section 3 describes the details of the proposed strategy optimization algorithm for white-box RGPO jamming. Section 4 presents numerical experiments validating the performance of the proposed algorithm. Section 5 concludes with a summary of this paper and a discussion of future work.

## 2. Problem Statement

### 2.1. Description of RGPO Jamming Process

**Figure 1:**
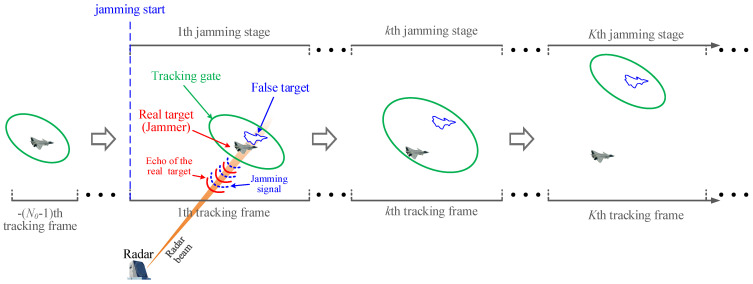
Diagram of RGPO jamming.

Assume that a radar has detected a target and has tracked it for $N_{0}$ frames. Now, as shown in Figure 1, the airborne jammer identified the threat and decided to perform RGPO jamming for countering the threat radar. In each jamming stage, the jammer begins by capturing the transmitted signal from the threat radar. This signal is then retransmitted to the receiver of the radar with a deliberately manipulated delay. As a result of this jamming signal, the threat radar perceives a false target appearing at a greater distance.

Suppose the RGPO jamming lasts *K* stages, and the controlled delay of the jamming signal at the *k*th jamming stage, $\tau_{k}$, is set as

(1) $$\begin{array}{c} \tau_{k}=\sum_{\tilde{k}=1}^{k}\Delta \tau_{\tilde{k}},k=1,2,\cdots ,K, \end{array}$$

where $\Delta \tau_{\tilde{k}}$ is the increment of the controlled delay at the $\tilde{k}$th jamming stage. Then, the distance, $d_{k}$, between the false target and the real target can be represented as

(2) $$\begin{array}{c} d_{k}=\frac{c\tau_{k}}{2}=\sum_{\tilde{k}=1}^{k}\frac{c\Delta \tau_{\tilde{k}}}{2},k=1,2,\cdots ,K, \end{array}$$

where *c* is the speed of light.

Without loss of generality, we assume that the real target follows a nearly constant velocity (CV) model [28], and the state matrix $X_{r}\left(k\right)$ is given by

(3) $$\begin{array}{c} X_{r}\left(k\right)=F_{r}\left(k-1\right)X_{r}\left(k-1\right)+U\left(k-1\right), \end{array}$$

where $F_{r}\left(k-1\right)$ is the state transition matrix and U(k−1) is the process noise. The matrix formulation of the above equation can be represented as

(4) $$\begin{array}{c} \left[\begin{array}{c} x_{r}^{\left(k\right)}\\ {\dot{x}}_{r}^{\left(k\right)}\\ y_{r}^{\left(k\right)}\\ {\dot{y}}_{r}^{\left(k\right)} \end{array}\right]=\left[\begin{array}{cccc} 1 & t & 0 & 0\\ 0 & 1 & 0 & 0\\ 0 & 0 & 1 & t\\ 0 & 0 & 0 & 1 \end{array}\right]\left[\begin{array}{c} x_{r}^{\left(k-1\right)}\\ {\dot{x}}_{r}^{\left(k-1\right)}\\ y_{r}^{\left(k-1\right)}\\ {\dot{y}}_{r}^{\left(k-1\right)} \end{array}\right]+\left[\begin{array}{c} 0.5\varepsilon_{x}^{\left(k\right)}t^{2}\\ \varepsilon_{x}^{\left(k\right)}t\\ 0.5\varepsilon_{y}^{\left(k\right)}t^{2}\\ \varepsilon_{y}^{\left(k\right)}t \end{array}\right], \end{array}$$

where $\left(x_{r}^{\left(k\right)},y_{r}^{\left(k\right)}\right)$ denotes the position coordinates of the real target at the *k*th jamming stage in the x-axis and y-axis, $\left({\dot{x}}_{r}^{\left(k\right)},{\dot{y}}_{r}^{\left(k\right)}\right)$ represents the velocities of the real target, *t* is the time interval between two successive jamming stages, and $\varepsilon_{x}^{\left(k\right)}$ and $\varepsilon_{y}^{\left(k\right)}$ denote the random variation of velocities of the real target.

Thus, the state matrix of the false target, $X_{f}\left(k\right)$, can be expressed as

(5) $$\begin{array}{c} X_{f}\left(k\right)=X_{r}\left(k\right)+\Theta \left(k\right)d_{k}, \end{array}$$

where Θ(k) is the coordinate transformation matrix. Corresponding to Equation (4), the matrix formulation of the above equation becomes

(6) $$\begin{array}{c} \left[\begin{array}{c} x_{f}^{\left(k\right)}\\ {\dot{x}}_{f}^{\left(k\right)}\\ y_{f}^{\left(k\right)}\\ {\dot{y}}_{f}^{\left(k\right)} \end{array}\right]=\left[\begin{array}{c} x_{r}^{\left(k\right)}\\ {\dot{x}}_{r}^{\left(k\right)}\\ y_{r}^{\left(k\right)}\\ {\dot{y}}_{r}^{k} \end{array}\right]+\left[\begin{array}{c} \cos(\theta_{k})\\ 0\\ \sin(\theta_{k})\\ 0 \end{array}\right]d_{k}, \end{array}$$

where $\left(x_{f}^{\left(k\right)},y_{f}^{\left(k\right)}\right)$ represents the position coordinates of the false target, $\left({\dot{x}}_{f}^{\left(k\right)},{\dot{y}}_{f}^{\left(k\right)}\right)$ represents the velocities of the false target, and $\theta_{k}$ is the azimuth angle of the real target and the false target.

Then, the measurement equation of the real target is given by

(7) $$\begin{array}{c} Z_{r}\left(k\right)=H\left(k\right)X_{r}\left(k\right)+W_{r}\left(k\right), \end{array}$$

where $Z_{r}\left(k\right)$ represents the measurement matrix of the real target, H(k) is the projection matrix that projects the state into the measurement space, and $W_{r}\left(k\right)$ represents the measurement noise of the real target. Corresponding to Equation (4), the matrix formulation of the above equation becomes

(8) $$\begin{array}{c} \left[\begin{array}{c} {\tilde{x}}_{r}^{\left(k\right)}\\ {\tilde{y}}_{r}^{\left(k\right)} \end{array}\right]=\left[\begin{array}{cccc} 1 & 0 & 0 & 0\\ 0 & 0 & 1 & 0 \end{array}\right]\left[\begin{array}{c} x_{r}^{\left(k\right)}\\ {\dot{x}}_{r}^{\left(k\right)}\\ y_{r}^{\left(k\right)}\\ {\dot{y}}_{r}^{k} \end{array}\right]+\left[\begin{array}{c} \delta_{r,x}^{\left(k\right)}\\ \delta_{r,y}^{\left(k\right)} \end{array}\right], \end{array}$$

where $\left({\tilde{x}}_{r}^{\left(k\right)},{\tilde{y}}_{r}^{\left(k\right)}\right)$ denotes the measurements of the position of the real target and $\delta_{r,x}^{\left(k\right)}$ and $\delta_{r,y}^{\left(k\right)}$ denote the random measurement error of position of the real target. Similarly, the measurement equation of the false target is given by

(9) $$\begin{array}{c} Z_{f}\left(k\right)=H\left(k\right)X_{f}\left(k\right)+W_{f}\left(k\right), \end{array}$$

where $Z_{f}\left(k\right)$ is the measurement matrix of the false target and $W_{f}\left(k\right)$ represents the measurement noise of the false target.

Next, the threat radar will receive the measurements $Z_{r}\left(k\right)$ and $Z_{f}\left(k\right)$ as input data. In the following, we will introduce the impact of $Z_{f}\left(k\right)$ on the threat radar in detail.

Radar tracking is a vast field with many branches, and researchers have conducted extensive research on each of them. The specific effect of the RGPO jamming differs slightly for different radar tracking approaches. Without loss of generality, a typical radar tracking system using the Kalman filter-probabilistic data association (KF-PDA) approach [29] is chosen as the tracking system of the threat radar in this subsection to illustrate the impact of the RGPO jamming. For this tracking system of the threat radar, the prediction equations are

(10) $$\begin{array}{cc} X(k|k-1)= & F(k-1)X(k-1), \end{array}$$

(11) $$\begin{array}{cc} Z(k|k-1)= & H(k)X(k|k-1), \end{array}$$

where X(k−1) is the target state estimation, X(k|k−1) is the one-step prediction of the threat radar for the target state, F(k−1) is the state transition matrix of the Kalman filter (KF) model, and Z(k|k−1) is the one-step prediction of the target measurement. The covariance matrix of the state prediction, P(k|k−1), is given by

(12) $$\begin{array}{c} P\left(k|k-1\right)=F\left(k-1\right)P\left(k-1\right)F^{T}\left(k-1\right)+Q_{p}\left(k-1\right), \end{array}$$

where P(k−1) is the covariance of the state estimation and $Q_{p}\left(k-1\right)$ is the covariance matrix of the process noise of the KF model.

Next, the tracking gate (also called the validation region) is built with Z(k|k−1) as the center to verify radar measurements. The typical tracking gate is the elliptical region [29], which can be expressed as

(13) $$\begin{array}{c} 𝒱\left(k,\gamma \right)=\left\{Z\left|{\left[Z-Z(k|k-1)\right]}^{T}S^{-1}\left(k\right)\left[Z-Z(k|k-1)\right]\leq \gamma \right.\right\}, \end{array}$$

where γ is the gate threshold and S(k) is the covariance matrix of the innovation, which can be given by

(14) $$\begin{array}{cc} S(k) & =H\left(k\right)P\left(k|k-1\right)H^{T}\left(k\right)+Q_{m}\left(k\right). \end{array}$$

where $Q_{m}\left(k\right)$ is the covariance matrix of the measurement noise. Measurements within the tracking gate are called the validated measurements.

Then, probabilistic data association (PDA) is performed to determine the weights (i.e., the association probabilities) of these validated measurements to participate in the tracking update.

**Figure 2:**
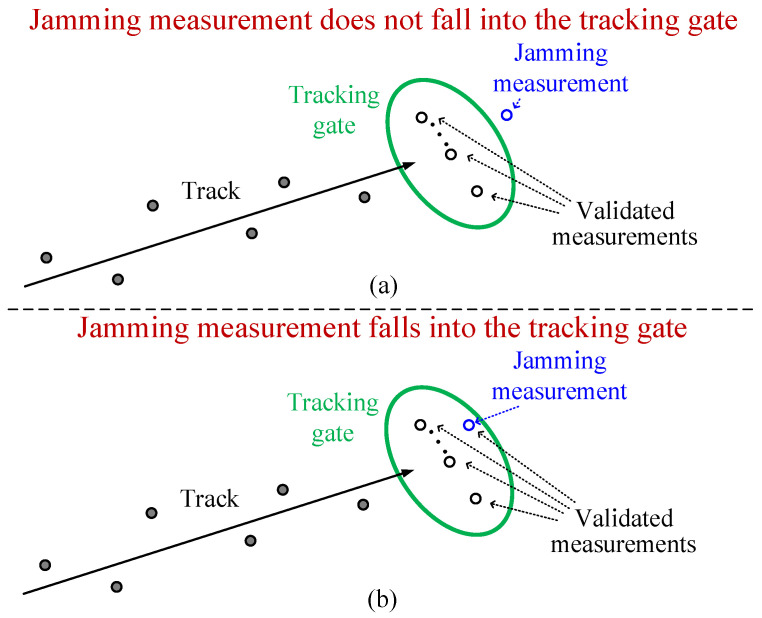
Schematic diagram of measurement verification.

Assume that the number of validated measurements at the *k*th jamming stage is $I_{k}$, which can be further expressed as

(15) $$I_{k}=\left\{\begin{array}{cc} I_{k}+1, & Z_{f}\left(k\right)\in 𝒱\left(k,\gamma \right)\\ I_{k}, & else \end{array}\right.,$$

where $I_{k}$ indicates the number of validated measurements without considering the RGPO jamming. If the jamming measurement $Z_{f}\left(k\right)$ does not fall into the tracking gate as shown in Figure 2a, it is logical to get $I_{k}=I_{k}$. Conversely, if $Z_{f}\left(k\right)$ falls into the tracking gate as shown in Figure 2b, $I_{k}=I_{k}+1$. It could be assumed that $Z_{f}\left(k\right)$ is the $\left(I_{k}+1\right)$th validated measurement.

Obviously, the measurement of the false target has a negative impact on the data association. The innovation, $v_{i}\left(k\right)$, of the *i*th validated measurement can be expressed as

(16) $$\begin{array}{c} v_{i}\left(k\right)=Z_{i}\left(k\right)-Z\left(k|k-1\right),i=1,2,\cdots ,I_{k} \end{array}$$

where $Z_{i}\left(k\right)$ represents the *i*th validated measurement at the *k*th jamming stage and $Z_{I_{k}+1}\left(k\right)=Z_{f}\left(k\right)$. The association probability of each validated measurement is calculated as follows [30]:

(17) $$\begin{array}{cc} \beta_{0}^{k} & =\frac{b}{b+\sum_{i=1}^{I_{k}}e_{i}}\\ & =\left\{\begin{array}{cc} \frac{b}{b+\sum_{i=1}^{I_{k}+1}e_{i}}, & Z_{f}\left(k\right)\in 𝒱\left(k,\gamma \right)\\ \frac{b}{b+\sum_{i=1}^{I_{k}}e_{i}}, & else \end{array}\right., \end{array}$$

(18) $$\begin{array}{cc} \beta_{i}^{k} & =\frac{e_{i}}{b+\sum_{\tilde{i}=1}^{I_{k}}e_{\tilde{i}}}.\\ & =\left\{\begin{array}{cc} \frac{e_{i}}{b+\sum_{\tilde{i}=1}^{I_{k}+1}e_{\tilde{i}}}, & Z_{f}\left(k\right)\in 𝒱\left(k,\gamma \right)\\ \frac{e_{i}}{b+\sum_{\tilde{i}=1}^{I_{k}}e_{\tilde{i}}}, & else \end{array}\right.,\\ \; & i=1,2,\cdots ,I_{k}, \end{array}$$

where

(19) $$\begin{array}{c} e_{i}=\exp\left\{-\frac{1}{2}v_{i}^{T}\left(k\right)S^{-1}\left(k\right)v_{i}\left(k\right)\right\}, \end{array}$$

(20) $$\begin{array}{c} b=\lambda {\left|2\pi S(k)\right|}^{\frac{1}{2}}\left(1-P_{D}P_{G}\right)/P_{D}, \end{array}$$

where $\beta_{0}^{k}$ represents the probability that there is no validated measurement derived from the real target, $\beta_{i}^{k}$ represents the association probability of *i*th validated measurement, and $\sum_{i=0}^{I_{k}}\beta_{i}^{k}=1$. $P_{D}$ denotes the target detection probability, $P_{G}$ is a parameter that accounts for restricting the normal density to the tracking gate, and λ is a parameter indicating the spatial density of false measurements.

From Equation (18), it can be seen that once the false measurement, $Z_{f}\left(k\right)$, falls into the tracking gate, it will be assigned the weight $\beta_{I_{k}+1}^{k}$. Compared with the case that false target measurement $Z_{f}\left(k\right)$ is not in the tracking gate, the proportion of the first $I_{k}$ weights within the set $\left\{\beta_{0}^{k},\beta_{1}^{k},\cdots ,\beta_{I_{k}+1}^{k}\right\}$ will be decreased, with the constraint $\sum_{i=0}^{I_{k}}\beta_{i}^{k}=1$. According to Equation (21), the combined innovation, v(k), will contain the wrong distance information carried by $Z_{f}\left(k\right)$, while the weight of the information carried by the real target will be decreased. This means that the combined innovation, v(k), tends to be closer to the innovation of the false target, $v_{I_{k}+1}\left(k\right)$, and away from the innovation of the real target. As a result, v(k) will tend to appear biased.

(21) $$\begin{array}{cc} v(k) & =\sum_{i=1}^{I_{k}}\beta_{i}\left(k\right)v_{i}\left(k\right)\\ & =\left\{\begin{array}{cc} \sum_{i=1}^{I_{k}+1}\beta_{i}^{k}v_{i}\left(k\right), & Z_{f}\left(k\right)\in 𝒱\left(k,\gamma \right)\\ \sum_{i=1}^{I_{k}}\beta_{i}^{k}v_{i}\left(k\right), & else \end{array}\right.. \end{array}$$

Finally, the combined innovation, v(k), is used to update the state estimation, and the estimation of the target state, X(k), is given by

(22) X(k)=X(k|k−1)+G(k)v(k),

where G(k) is the KF gain and

(23) $$G\left(k\right)=P\left(k|k-1\right)H^{T}\left(k\right)S^{-1}\left(k\right).$$

As shown in Equation (22), the error derived from $Z_{f}\left(k\right)$ will be transmitted to X(k) via v(k), resulting in an error in the estimation of the target state. Specifically, the estimate of the target state will be closer to the false target measurement, $Z_{f}\left(k\right)$, and farther away from the real target measurement, $Z_{r}\left(k\right)$. Similarly, the error component will also be transmitted to P(k) via v(k), as shown in Equations (24)–(27).

(24) $$\begin{array}{cc} P(k)= & P\left(k|k-1\right)\beta_{0}^{k}+\left[1-\beta_{0}^{k}\right]P^{c}\left(k\right)+\tilde{P}\left(k\right), \end{array}$$

(25) $$\begin{array}{cc} P^{c}\left(k\right) & =\left[I-G(k)H(k)\right]P\left(k|k-1\right), \end{array}$$

(26) $$\begin{array}{cc} \tilde{P}\left(k\right) & =G\left(k\right)\left[B\left(k\right)-v\left(k\right)v^{T}\left(k\right)\right]G^{T}\left(k\right), \end{array}$$

(27) $$\begin{array}{cc} B(k) & =\sum_{i=1}^{I_{k}}\beta_{i}^{k}v_{i}\left(k\right)v_{i}^{T}\left(k\right)\\ & =\left\{\begin{array}{cc} \sum_{i=1}^{I_{k}+1}\beta_{i}^{k}v_{i}\left(k\right)v_{i}^{T}\left(k\right), & Z_{f}\left(k\right)\in 𝒱\left(k,\gamma \right)\\ \sum_{i=1}^{I_{k}}\beta_{i}^{k}v_{i}\left(k\right)v_{i}^{T}\left(k\right), & else \end{array}\right.. \end{array}$$

Moreover, the negative impact of $Z_{f}\left(k\right)$ is not limited to the current *k*th jamming stage; X(k) and P(k) will be used for the prediction of the state and measurement to the (k+1)th jamming stage, as shown in Equations (10) and (12). Correspondingly, the one-step prediction of the target state, X(k+1|k), and the measurement, Z(k+1|k), of the (k+1)th jamming stage are also influenced by the error component due to $Z_{f}\left(k\right)$. As a result, the center of the tracking gate, Z(k+1|k), is shifted away from the measurement of the real target, $Z_{r}\left(k+1\right)$. In the case described above, if the false target’s measurement at the (k+1)th jamming stage remains within the tracking gate, the tracking error will increase further, leading to a continued shift in the tracking gate. Moreover, when the controlled delays across successive tracking frames are properly coordinated, the tracking error can rapidly compound, causing the tracking gate to significantly diverge from the real target.

Although there are many different approaches to radar tracking, the principle of the RGPO jamming is basically similar: use the measurements of false target to confuse the data association of the tracking system of the threat radar, thereby affecting the target state estimation. Therefore, it is very important to set the jamming strategy properly.

### 2.2. Optimization Model of White-Box RGPO Jamming Strategy

**Figure 3:**
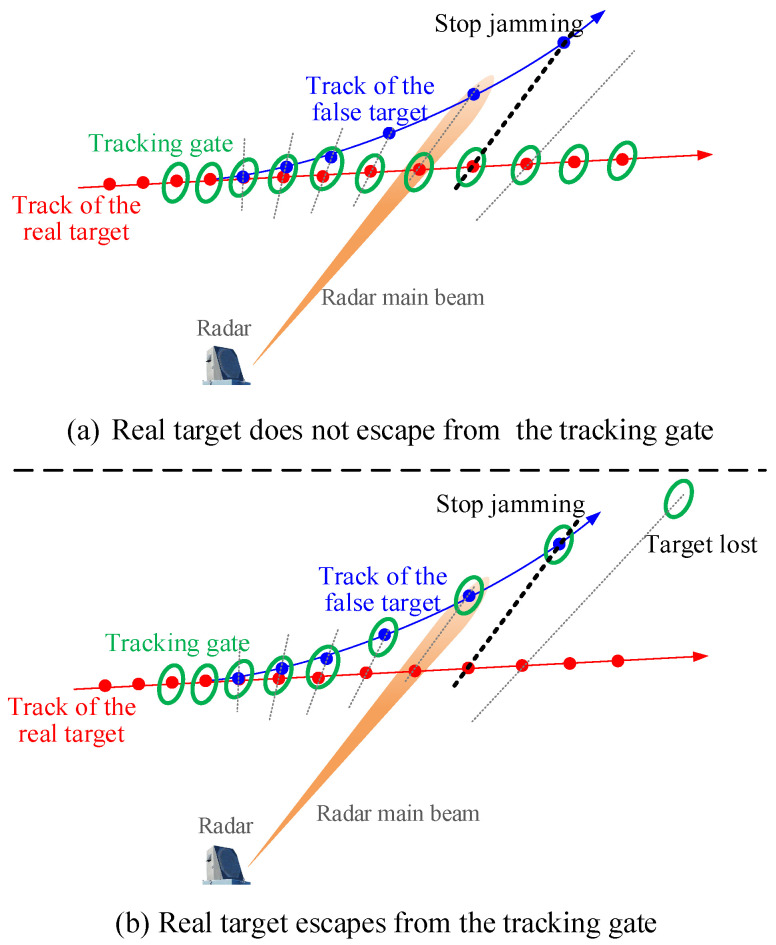
Schematic diagram of jamming result.

For RGPO jamming, the quality of the jamming results is mainly evaluated by the following two points [10]:

1. Whether or not the real target escapes from the tracking gate: If the real target does not escape from the tracking gate, as shown in Figure 3a, then the jamming effect is very limited. On the contrary, if the measurement of the real target escapes from the tracking gate, as shown in Figure 3b, this means that the threat radar has temporarily lost the real target.
2. The miss distance: In the case of a larger miss distance, the real target is more difficult to recaptured by the tracking gate, and the real target is safer.

For a specific sampling of the jamming strategy φ, $\xi_{\varphi }^{\left(K\right)}$ is used to denote whether or not the real target escapes from the tracking gate, which is given by

(28) $$\xi_{\varphi }^{\left(K\right)}=\left\{\begin{array}{cc} 0, & Z_{r}\left(K\right)\in 𝒱\left(K,\gamma \right)\\ 1, & else \end{array}\right.,$$

where $\xi_{\varphi }^{\left(K\right)}=1$ signifies that the real target has moved outside the tracking gate, whereas $\xi_{\varphi }^{\left(K\right)}=0$ indicates that the real target remains inside the tracking gate. Moreover, for a specific sampling of the jamming strategy φ, the corresponding miss distance can be expressed as

(29) $$\begin{array}{c} {\mathrm{MD}}_{\varphi }^{\left(K\right)}=\sqrt{{\left[x^{\left(K\right)}-x_{r}^{\left(K\right)}\right]}^{2}+{\left[y^{\left(K\right)}-y_{r}^{\left(K\right)}\right]}^{2}}, \end{array}$$

where $\left(x^{\left(K\right)},y^{\left(K\right)}\right)$ denotes the position estimation of the real target and $\left(x_{r}^{\left(K\right)},y_{r}^{\left(K\right)}\right)$ represents the actual position of the real target.

Assume the threat radar tracking model is *m*. Then, for a particular sampling of the jamming strategy φ, the quality of jamming result can be evaluated as

(30) $$\begin{array}{c} \left[\xi_{\varphi }^{\left(K\right)},{\mathrm{MD}}_{\varphi }^{\left(K\right)}\right]=\left[r_{1}\left(\varphi ,𝒳,𝒲,m\right),r_{2}\left(\varphi ,𝒳,𝒲,m\right)\right], \end{array}$$

where $r_{1}\left(\cdot \right)$ and $r_{2}\left(\cdot \right)$ denote the objective functions, $\varphi =[\Delta \tau_{1},\Delta \tau_{2},\cdots$, $\Delta \tau_{K}]$ represents the increment of the controlled delay of the jamming signal for the *K* jamming stages, $𝒳=[X_{r}\left(1\right),X_{r}\left(2\right),\cdots$, $X_{r}\left(K\right)]$ represents the state of the real target with process noise for the *K* jamming stages, and $𝒲=[W_{r}\left(1\right),W_{r}\left(2\right),\cdots$, $W_{r}\left(K\right),W_{f}\left(1\right),W_{f}\left(2\right),\cdots$, $W_{f}\left(K\right)]$ represents the measurement noise of the *K* jamming stages.

Due to the fact that 𝒳 and 𝒲 are random variables, $\xi_{\varphi }^{\left(K\right)}$ and ${\mathrm{MD}}_{\varphi }^{\left(K\right)}$ are indeterminate. Therefore, jamming strategy φ cannot be accurately evaluated only by the jamming result of a particular sampling. In this paper, we consider evaluating the performance of the jamming strategy using the expected values of $\xi_{\varphi }^{\left(K\right)}$ and ${\mathrm{MD}}_{\varphi }^{\left(K\right)}$. Thus, the strategy optimization problem for RGPO jamming can be expressed as

(31) $$\max_{\varphi }\;\left[E\left[\xi_{\varphi }^{\left(K\right)}\right],E\left[{\mathrm{MD}}_{\varphi }^{\left(K\right)}\right]\right]=\left[E\left[r_{1}\left(\varphi ,𝒳,𝒲,m\right)\right],E\left[r_{2}\left(\varphi ,𝒳,𝒲,m\right)\right]\right]\;s.t.\varphi \in \Omega ,\;\;\;$$

where E[·] represents expected value, while Ω indicates solution space. Because of the conflicting nature of the two objective functions in problem (31), achieving a global maximum for all objectives simultaneously at a single solution is unfeasible. The weighted aggregation method is widely recognized as a standard technique for handling multi-objective optimization problems [31,32,33]. Using this method, the score corresponding to a specific sample of the strategy φ is determined as follows:

(32) $$\begin{array}{c} r\left(\varphi \right)=w_{1}\cdot \xi_{\varphi }^{\left(K\right)}+w_{2}\cdot {\mathrm{MD}}_{\varphi }^{\left(K\right)}, \end{array}$$

where $w_{1}>0$ and $w_{2}>0$ represent the respective weights assigned to the objective functions. Using this scoring framework, the strategy optimization problem for RGPO jamming can be further expressed as

(33) $$\begin{array}{cc} \max_{\varphi }E\left[r(\varphi )\right] & =w_{1}\cdot E\left[\xi_{\varphi }^{\left(K\right)}\right]+w_{2}\cdot E\left[{\mathrm{MD}}_{\varphi }^{\left(K\right)}\right]\\ & =w_{1}\cdot E\left[r_{1}\left(\varphi ,𝒳,𝒲,m\right)\right]+w_{2}\cdot E\left[r_{2}\left(\varphi ,𝒳,𝒲,m\right)\right]\\ s.t. & \varphi \in \Omega . \end{array}$$

Because of the high complexity of RGPO jamming, it is challenging to derive algebraic expressions for the objective functions $r_{1}\left(\cdot \right)$ and $r_{2}\left(\cdot \right)$. Moreover, the jammer cannot directly observe the jamming outcomes, such as $\xi_{\varphi }^{\left(K\right)}$ and ${\mathrm{MD}}_{\varphi }^{\left(K\right)}$, from the threat radar. Additionally, the presence of noise introduces uncertainty into the jamming results. These three factors collectively render the jamming strategy optimization problem, as shown in Equation (33), an ASSO problem. Unlike standard SSO problems, the primary difficulty in ASSO problems lies in obtaining the necessary input–output data. However, in white-box scenarios, the knowledge of the tracking radar is available. This enables the construction of a local simulation for the tracking radar, which will be used to approximate the jamming outcomes. Consequently, it becomes feasible to address the above jamming strategy optimization problem using SSO algorithms assisted by local simulation.

## 3. White-Box RGPO Jamming Strategy Generation

### 3.1. Overall Framework

**Figure 4:**
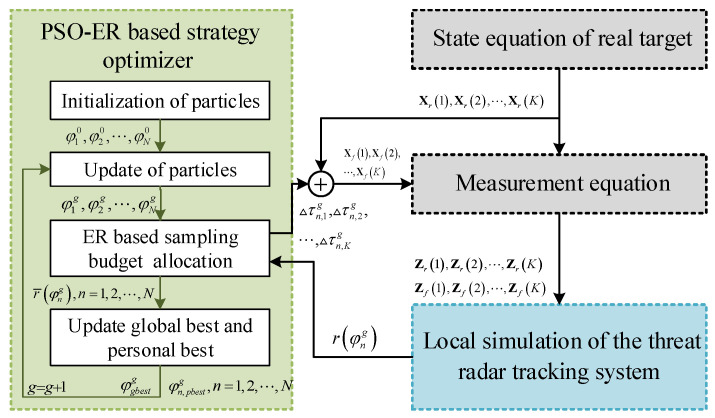
Generic diagram of the white-box RGPO jamming strategy optimization.

In this section, we describe the process of designing a white-box RGPO jamming strategy. As illustrated in Figure 4, the PSO-ER algorithm functions as the optimizer for the jamming strategy. The PSO-ER combines PSO with ER, where PSO explores the solution domain and produces candidate strategies to be evaluated. To reduce the error in the comparison of candidate strategies caused by noise, we estimate their fitness (i.e., the jamming performance) using the ER scheme. In the ER, all candidate strategies are assigned the same number of resampling opportunities. For each resampling, the jamming result is obtained by the local simulation of the tracking radar. Finally, the fitness of the candidate strategies are estimated as the mean of the jamming results of the multiple resampling. Below, the process of the strategy optimization is introduced in detail.

### 3.2. PSO-ER-Based Strategy Optimizer

In the PSO-ER framework, a group of particles moves through the decision space, iteratively converging toward the optimal strategy. Each particle is characterized by its position and velocity, where the position represents a potential candidate strategy. The optimization procedure is carried out in three fundamental stages.

Firstly, we initialize the positions $\varphi_{n}^{0}=[\Delta \tau_{n1}^{0},\Delta \tau_{n2}^{0},\cdots$, $\Delta \tau_{nK}^{0}],n=1,2,\cdots$, *N* and velocities $\upsilon_{n}^{0}=[\upsilon_{n1}^{0},\upsilon_{n2}^{0},\cdots$, $\upsilon_{nK}^{0}],n=1,2,\cdots$, *N* of *N* particles using Latin hypercube sampling (LHS). Following the initialization process, the fitness of all particles (i.e., the performance of the associated candidate strategies) is evaluated using the ER scheme. Within this scheme, *J* resamples are conducted for each candidate strategy, and their performance is estimated by

(34) $$\begin{array}{c} \bar{r}\left(\varphi_{n}\right)=w_{1}\cdot {\bar{\xi }}_{\varphi_{n}}^{\left(K\right)}+w_{2}\cdot {\bar{\mathrm{MD}}}_{\varphi_{n}}^{\left(K\right)},n=1,2,\cdots ,N, \end{array}$$

where $\bar{r}\left(\varphi_{n}\right)$ denotes the average score obtained by *J* resamples of the candidate strategy $\varphi_{n}$, ${\bar{\xi }}_{\varphi_{n}}^{\left(K\right)}$ denotes the ratio of the real target escaping from the tracking gate in *J* resamples of the candidate strategy $\varphi_{n}$, and ${\bar{\mathrm{MD}}}_{\varphi_{n}}^{\left(K\right)}$ denotes the average miss distance obtained by *J* resamples of the candidate strategy $\varphi_{n}$.

Then, for each iteration, the velocities and positions of all particles are updated as follows:

(35) $$\begin{array}{c} \upsilon_{n}^{g}=\alpha^{g}\upsilon_{n}^{g-1}+c_{1}\delta_{1}^{g}\left(\varphi_{n,\mathrm{pbest}}^{g-1}-\varphi_{n}^{g-1}\right)+c_{2}\delta_{2}^{g}\left(\varphi_{\mathrm{gbest}}^{g-1}-\varphi_{n}^{g-1}\right),n=1,2,\cdots ,N, \end{array}$$

(36) $$\begin{array}{c} \varphi_{n}^{g}=\varphi_{n}^{g-1}+\upsilon_{n}^{g},n=1,2,\cdots ,N, \end{array}$$

where $\upsilon_{n}^{g}$ and $\varphi_{n}^{g}$ respectively denote the current velocity and position of the *n*th particle at the *g*th iteration, $\varphi_{n,\mathrm{pbest}}^{g-1}$ is the personal best position of the *n*th particle at the (g−1)th iteration (i.e., the best jamming strategy found by the *n*th particle so far, and the initial personal best position $\varphi_{n,\mathrm{pbest}}^{0}$ is set to be equal as $\varphi_{n}^{0}$), $\varphi_{\mathrm{gbest}}^{g-1}$ is the global best position at the (g−1)th iteration (i.e., the best jamming strategy found by all particles so far). The sequences $\delta_{1}$ and $\delta_{2}$ are *K*-dimensional, with each dimension being a random value in the range [0, 1]. As recommended in [34], the constants $c_{1}$ and $c_{2}$ are both assigned a value of 1.49445. The inertia weight, denoted as $\alpha^{g}$, governs velocity updates and decreases linearly from 0.9 to 0.4 as iteration *g* progresses, according to (37), where $g_{\max}$ represents the maximum number of iterations [35].

(37) $$\alpha^{g}=0.9-0.5\frac{g}{g_{\max}}.$$

Finally, we recalculated the fitness of each particle using the ER scheme and update the personal best position, $\varphi_{n,\mathrm{pbest}}^{g}$, and the global best position, $\varphi_{\mathrm{gbest}}^{g}$, as follows:

(38) $$\varphi_{n,\mathrm{pbest}}^{g}=\left\{\begin{array}{cc} \varphi_{n}^{g}, & \bar{r}\left(\varphi_{n}^{g}\right)>\bar{r}\left(\varphi_{n,\mathrm{pbest}}^{g-1}\right)\\ \varphi_{n,\mathrm{pbest}}^{g-1}, & else \end{array}\right.,$$

(39) $$\begin{array}{c} \varphi_{\mathrm{gbest}}^{g}=\arg\max_{\varphi_{n,\mathrm{pbest}}^{g}}\bar{r}\left(\varphi_{n,\mathrm{pbest}}^{g}\right). \end{array}$$

Thus far, the optimal jamming strategy at the *g*th iteration can be derived from $\varphi_{\mathrm{gbest}}^{g}$.

The above iteration process will persist until the preset maximum number of iterations, $g_{\max}$, is satisfied, at which point the final RGPO jamming strategy, $\varphi_{\mathrm{gbest}}^{g_{\max}}$, will be produced. A concise overview of the proposed strategy optimization algorithm for white-box RGPO jamming is given in Algorithm 1.
**Algorithm 1** Local simulation-assisted PSO-ER-based white-box RGPO jamming strategy optimization algorithm.**Input:** The motion model of the real target. The local simulation of the threat radar tracking system. Preset parameters: *K*, *N*, *J*, $g_{\max}$., etc;1:Initialize the positions $\varphi_{n}^{0},n=1,2,\cdots ,N$ and the velocities $\upsilon_{n}^{0},n=1,2,\cdots ,N$ of particles using LHS;2:Estimate the fitness $\bar{r}\left(\varphi_{n}^{0}\right),n=1,2,\cdots ,N$ of particles by the ER scheme as Equation (32);3:Initialize the personal best position $\varphi_{n,\mathrm{pbest}}^{0},n=1,2,\cdots ,N$ and the global best position $\varphi_{\mathrm{gbest}}^{0}$;4:**for** $g=1:g_{\max}$ **do**5:      Calculate the inertia weight $\alpha^{g}$ using (37);6:      Update the velocities $\upsilon_{n}^{g},n=1,2,\cdots ,N$ and the positions $\varphi_{n}^{g},n=1,2,\cdots ,N$ of particles using Equations (35) and (36);7:      Estimate the fitness $\bar{r}\left(\varphi_{n}^{g}\right),n=1,2,\cdots ,N$ of particles by the ER scheme as Equation (32);8:      Update the personal best position of *N* particles $\varphi_{n,\mathrm{pbest}}^{g},n=1,2,\cdots ,N$ and the global best position $\varphi_{\mathrm{gbest}}^{g}$ using Equations (38) and (39);9:**end for****Output:** The jamming strategy, $\varphi_{\mathrm{gbest}}^{g_{\max}}$;

## 4. Empirical Study

From Equation (33), we can see that the performance of the RGPO jamming strategy is related to the motion model of the real target and the tracking model of the threat radar. To evaluate the performance of the proposed method on different motion models and tracking models, four typical tracking problems from [10] are chosen as the benchmark problems, and their parameters are shown in Table 1.

**Remark** **1.** *It should be mentioned that, before the jamming, the threat radar has established a track for $N_{0}$ tracking frames, as shown in Figure 1. However, in practice, $N_{0}$ is not fixed and depends on the response time, $T_{0}$, of the jammer to the threat. Generally, the value of $T_{0}$ should meet the following two points: (1) The identification of the threat by the jammer requires time to intercept enough information, so a small $T_{0}$ is a small probability event; (2) When the jammer intercepts enough information, the probability of missing detection of a threat should be extremely low, so a large $T_{0}$ is also a small probability event. Therefore, it is not difficult to find that $T_{0}$ should follow an approximate Gaussian distribution. Without loss of generality, the value of $T_{0}$ can be given by*

(40) $$T_{0}=\left\{\begin{array}{cc} T_{\mathrm{temp}}, & T_{\mathrm{temp}}\geq 0\\ 0, & T_{\mathrm{temp}}<0 \end{array}\right.,$$

*where $T_{\mathrm{temp}}$ denotes a temporary variable, which follows a Gaussian distribution with mean $T_{e}$ and variance $\sigma_{e}^{2}$. Then, $N_{0}$ can be obtained by*

(41) $$\begin{array}{c} N_{0}=\left\lceil \frac{T_{0}}{t}\right\rceil . \end{array}$$

*Since the setting of $N_{0}$ is not the focus of this paper, we set $T_{e}=5,\sigma_{e}^{2}=2$ in the following experiments.*

**Table 1:** Parameter settings.

Radar Parameters	
Time interval *t*	1s
Measurement noise	Range: Gaussian of $N(0,10^{2})$; Angle: Gaussian of $N(0,0.00175^{2})$
Detection probability	0.99
Elliptic gate parameter γ	16
**CV Benchmark**	
Target motion model	Nearly constant velocity (CV) model
Target initial state	$\left(x_{r}^{\left(0\right)},{\dot{x}}_{r}^{\left(0\right)},y_{r}^{\left(0\right)},{\dot{y}}_{r}^{\left(0\right)}\right)$=(50 km,50 m/s,55 km,350 m/s)
Target motion process noise	x axis: Gaussian of $N(0,5^{2})$; y axis: Gaussian of $N(0,5^{2})$
Tracking method	KF-PDA
Process noise of KF model	x axis: Gaussian of $N(0,10^{2})$; y axis: Gaussian of $N(0,10^{2})$
**CA Benchmark**	
Target motion model	Nearly constant acceleration (CA) model
Target initial state	$\left(x_{r}^{\left(0\right)},{\dot{x}}_{r}^{\left(0\right)},{\ddot{x}}_{r}^{\left(0\right)},y_{r}^{\left(0\right)},{\dot{y}}_{r}^{\left(0\right)},{\ddot{y}}_{r}^{\left(0\right)}\right)$=$\left(50\mathrm{km},25m/s,10m/s^{2},55\mathrm{km},350m/s,10m/s^{2}\right)$
Target motion process noise	x axis: Gaussian of $N(0,1^{2})$; y axis: Gaussian of $N(0,1^{2})$
Tracking method	KF-PDA
Process noise of KF model	x axis: Gaussian of $N(0,2^{2})$; y axis: Gaussian of $N(0,2^{2})$
**CT Benchmark**	
Target motion model	Nearly coordinate turn (CT) model
Target initial state	$\left(x_{r}^{\left(0\right)},{\dot{x}}_{r}^{\left(0\right)},y_{r}^{\left(0\right)},{\dot{y}}_{r}^{\left(0\right)},\omega \right)$=(50km,25m/s,55km,350m/s,0.15rad/s)
Target motion process noise	x axis: Gaussian of $N(0,5^{2})$; y axis: Gaussian of $N(0,5^{2})$
Tracking method	KF-PDA
Process noise of KF model	x axis: Gaussian of $N(0,10^{2})$; y axis: Gaussian of $N(0,10^{2})$
**RV Benchmark**	
Target motion model	Nearly exoatmospheric re-entry vehicle (RV) model
Target initial state	$\left(x_{r}^{\left(0\right)},{\dot{x}}_{r}^{\left(0\right)},y_{r}^{\left(0\right)},{\dot{y}}_{r}^{\left(0\right)}\right)$=(48.5km,−1720m/s,22km,−946m/s)
Target motion process noise	x axis: Gaussian of $N(0,5^{2})$; y axis: Gaussian of $N(0,5^{2})$
Tracking method	EKF-PDA
Process noise of KF model	x axis: Gaussian of $N(0,10^{2})$; y axis: Gaussian of $N(0,10^{2})$
Ballistic parameters	40,000
Atmospheric density parameter	$\lambda_{1}=1.227;\;\;\lambda_{2}=1.093\times 10^{-4}$

$\left(x_{r}^{0},y_{r}^{0}\right)$ represents the initial position of the target; $\left({\dot{x}}_{r}^{\left(0\right)},{\dot{y}}_{r}^{\left(0\right)}\right)$ represents the velocity of the target; $\left({\ddot{x}}_{r}^{\left(0\right)},{\ddot{y}}_{r}^{\left(0\right)}\right)$ represents the acceleration of the target; ω represents the angular velocity of the target.

### 4.1. Parameter Sensitivity Analysis

To analyze how certain key parameters affect the performance of the proposed approach, we carried out a series of experiments on the CV benchmark problem. Moreover, to better verify the effectiveness, we compare the proposed method with UV-RGPO and UA-RGPO. However, the setting of parameters of UV-RGPO and UA-RGPO (i.e., the pull-off velocity, $v_{po}$, for the UV-RGPO, the initial pull-off velocity, $v_{ipo}$, and the pull-off acceleration, $a_{po}$, for the UA-RGPO) have an impact on their performance. To our best knowledge, there is no specific method in the open literature for setting these parameters. In practice, these parameters are set based on expert experience. In this paper, to better express the advantages of the proposed method, we find the performance of the best UV-RGPO and UA-RGPO strategies by traversal, then compare them with the average performance of strategies obtained by the proposed method.

#### 4.1.1. Scalability

To test how well the proposed method scales with an increasing number of jamming stages, *K*, the proposed method is compared with UV-RGPO and UA-RGPO on the CV benchmark with various *K*. To be fair, the weights of objective functions are set to be the same ($w_{1}=0.5,w_{2}=0.5/200$). To find the best UV-RGPO strategy, we traverse $v_{po}$ within $\left[v_{po}^{\min},v_{po}^{\max}\right]$=[2m/s,50m/s]. Similarly, to find the best UA-RGPO strategy, we traverse $v_{ipo}$ within $\left[v_{ipo}^{\min},v_{ipo}^{\max}\right]=\left[2\;m/s,50\;m/s\right]$ and $a_{po}$ within $\left[a_{po}^{\min},a_{po}^{\max}\right]=\left[2\;m/s^{2},20\;m/s^{2}\right]$. For the proposed method, 50 independent runs are performed, the PSO is initialized with N=40 particles, the number of resampling for each candidate strategy is J=2000, and the maximum number of iterations is $g_{\max}=100$.

**Figure 5:**
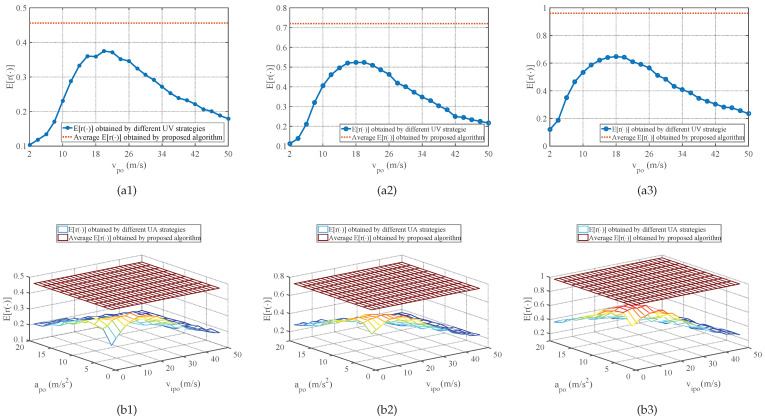
The average E[r(φ)] performance of strategies obtained by the proposed method in comparison with the E[r(φ)] performance of different UV-RGPO and UA-RGPO strategies on the CV benchmark with various *K*. (**a1**) Proposed method vs. UV-RGPO, K=10; (**b1**) Proposed method vs. UA-RGPO, K=10; (**a2**) Proposed method vs. UV-RGPO, K=15; (**b2**) Proposed method vs. UA-RGPO, K=15; (**a3**) Proposed method vs. UV-RGPO, K=20; (**b3**) Proposed method vs. UA-RGPO, K=20.

Figure 5 shows the average E[r(φ)] performance of strategies obtained by the proposed method in comparison with the E[r(φ)] performance of different UV-RGPO and UA-RGPO strategies on CV benchmarks with different *K*, where the E[r(φ)] is estimated by 10,000 independent Monte Carlo trials. Moreover, the average $E[\xi_{\varphi }^{\left(K\right)}]$, $E[{\mathrm{MD}}_{\varphi }^{\left(K\right)}]$ performances of strategies obtained by the proposed method, and the $E[\xi_{\varphi }^{\left(K\right)}]$, $E[{\mathrm{MD}}_{\varphi }^{\left(K\right)}]$ performances of the best UV-RGPO strategy and UA-RGPO strategy, are tabulated in Table 2. Similarly, the $E[\xi_{\varphi }^{\left(K\right)}]$ and $E[{\mathrm{MD}}_{\varphi }^{\left(K\right)}]$ are estimated by 10,000 independent Monte Carlo trials. From Figure 5, it can be seen that the E[r(φ)] performance of strategies obtained by the proposed method is significantly better than all the UV-RGPO and UA-RGPO strategies when *K* changes from 10 to 20, and the gain of the proposed method does not change much as the maximum number of jamming stages increases. The observation we can make from Table 2 is that the $E[\xi_{\varphi }^{\left(K\right)}]$ and $E[{\mathrm{MD}}_{\varphi }^{\left(K\right)}]$ performances of strategies obtained by the proposed method are better than the best UV-RGPO and UA-RGPO strategies when *K* changes from 10 to 20. These results prove that the proposed method is better than UV-RGPO and UA-RGPO and show that the proposed method has good scalability to the maximum number of jamming stages.

#### 4.1.2. Effect of Scoring Mechanism

To show the effect of the scoring mechanism on the balance of the $E[\xi_{\varphi }^{\left(K\right)}]$ and $E[{\mathrm{MD}}_{\varphi }^{\left(K\right)}]$ performances, we compare the proposed method with UV-RGPO and UA-RGPO on the CV benchmark using different objective function weights ($w_{1},w_{2}$). The total numbers of jamming stages is K=15. Similarly, we traverse $v_{po}$ within $\left[v_{po}^{\min},v_{po}^{\max}\right]$=[2m/s,50m/s] to find the best UV-RGPO strategy and traverse $v_{ipo}$ within $\left[v_{ipo}^{\min},v_{ipo}^{\max}\right]=\left[2\;m/s,50\;m/s\right]$ and $a_{po}$ within $\left[a_{po}^{\min},a_{po}^{\max}\right]=\left[2\;m/s^{2},20\;m/s^{2}\right]$ to find the best UA-RGPO strategy. For the proposed method, 50 independent runs are performed, the PSO is initialized with N=40 particles, the number of resampling for each candidate strategy is J=2000, and the maximum number of iterations is $g_{\max}=100$.

The average E[r(φ)], $E[\xi_{\varphi }^{\left(K\right)}]$, and $E[{\mathrm{MD}}_{\varphi }^{\left(K\right)}]$ performances of strategies obtained by the proposed method and the E[r(φ)], $E[\xi_{\varphi }^{\left(K\right)}]$, and $E[{\mathrm{MD}}_{\varphi }^{\left(K\right)}]$ performances of the best UV-RGPO strategy and UA-RGPO strategy are tabulated in Table 3. As shown in Table 3, with the increase of the weight $w_{1}$, the $E[\xi_{\varphi }^{\left(K\right)}]$ performance of these three methods gradually improves, and $E[{\mathrm{MD}}_{\varphi }^{\left(K\right)}]$ performance gradually decreases. That is because there is a certain degree of conflict between the $E[\xi_{\varphi }^{\left(K\right)}]$ and $E[{\mathrm{MD}}_{\varphi }^{\left(K\right)}]$ performances for the same RGPO jamming strategy. Therefore, when the weight $w_{1}$ is larger and the weight $w_{2}$ is smaller, the generated jamming strategies prefer the $E[\xi_{\varphi }^{\left(K\right)}]$ performance, and vice versa, and the generated jamming strategies prefer the $E[{\mathrm{MD}}_{\varphi }^{\left(K\right)}]$ performance. In practice, the weights $w_{1}$ and $w_{2}$ can be flexibly set according to task requirements. Another observation we can make is that the proposed method is better than UV-RGPO and UA-RGPO under different $w_{1}$ and $w_{2}$. This observation further suggests the effectiveness of the proposed method.

### 4.2. Experiments on Different Benchmark Problems

For a more comprehensive evaluation of the proposed method, we test the proposed method on the other three benchmark problems (CA, CT, and RV). The total number of jamming stages is K=15. To better verify the effectiveness, we compare the proposed method with UV-RGPO and the UA-RGPO. To be fair, the above three methods use the same settings for the weights of objective functions ($w_{1}=0.5,w_{2}=0.5/200$). For UV-RGPO, we traverse $v_{po}$ within $\left[v_{po}^{\min},v_{po}^{\max}\right]$ = [2m/s,50m/s] to find the best strategy. For UA-RGPO, we traverse $v_{ipo}$ within $\left[v_{ipo}^{\min},v_{ipo}^{\max}\right]=\left[2\;m/s,50\;m/s\right]$ and $a_{po}$ within $\left[a_{po}^{\min},a_{po}^{\max}\right]=\left[2\;m/s^{2},20\;m/s^{2}\right]$ to find the best strategy. For the proposed method, 50 independent runs are performed, the PSO is initialized with N=40 particles, the number of resampling for each candidate strategy is J=2000, and the maximum number of iterations is $g_{\max}=100$.

**Figure 6:**
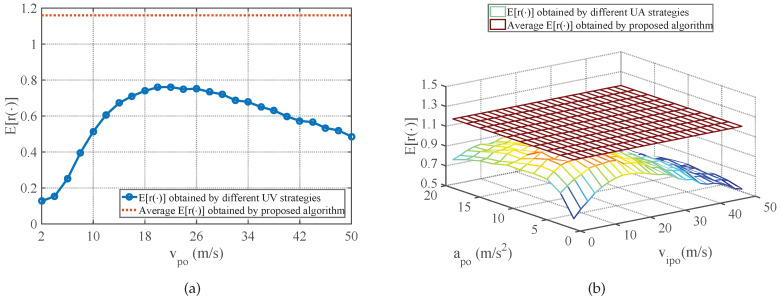
The average E[r(φ)] performance of strategies obtained by the proposed method in comparison with the E[r(φ)] performance of different UV-RGPO and UA-RGPO strategies on the CA benchmark. (**a**) Proposed method vs. UV-RGPO; (**b**) Proposed method vs. UA-RGPO.

**Figure 7:**
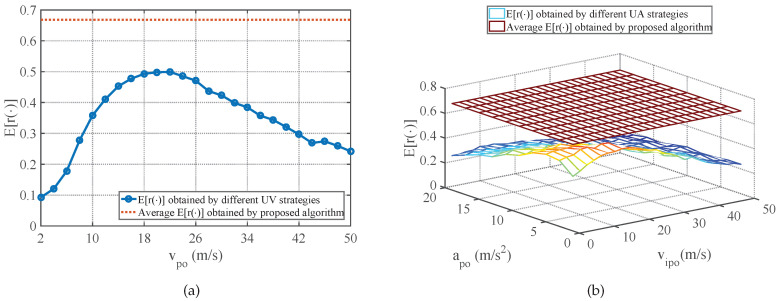
The average E[r(φ)] performance of strategies obtained by the proposed method in comparison with the E[r(φ)] performance of different UV-RGPO and UA-RGPO strategies on the CT benchmark. (**a**) Proposed method vs. UV-RGPO; (**b**) Proposed method vs. UA-RGPO.

**Figure 8:**
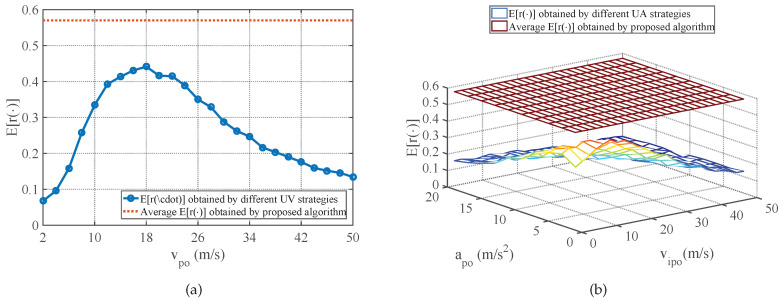
The average E[r(φ)] performance of strategies obtained by the proposed method in comparison with the E[r(φ)] performance of different UV-RGPO and UA-RGPO strategies on the RV benchmark. (**a**) Proposed method vs. UV-RGPO; (**b**) Proposed method vs. UA-RGPO.

The average E[r(φ)] performance of strategies obtained by the proposed method in comparison with the E[r(φ)] performance of different UV-RGPO and UA-RGPO strategies are shown in Figure 6, Figure 7 and Figure 8. Moreover, the average $E[\xi_{\varphi }^{\left(K\right)}]$ and $E[{\mathrm{MD}}_{\varphi }^{\left(K\right)}]$ performances of strategies obtained by the proposed method and the $E[\xi_{\varphi }^{\left(K\right)}]$ and $E[{\mathrm{MD}}_{\varphi }^{\left(K\right)}]$ performances of the best UV-RGPO strategy and UA-RGPO strategy are tabulated in Table 4. From these results, we clearly see that the proposed method outperforms UV-RGPO and UA-RGPO on all four benchmarks (including the CV benchmark), despite the fact that the motion models of the real target and the tracking models of the threat radar vary between the benchmarks. The numerical result shows that the proposed method is not sensitive to the motion models of the real target and the tracking models of the threat radar.

## 5. Conclusions

This paper addresses the strategy optimization problem for white-box RGPO jamming. The strategy optimization of the RGPO jamming has been formulated as an ASSO problem, and a white-box RGPO jamming strategy optimization algorithm based on PSO-ER with local simulation assistance has been presented. In this algorithm, PSO-ER is utilized to identify the optimal jamming strategy, while a locally constructed simulation of the tracking radar provides performance evaluations for candidate strategies. Experimental findings demonstrate that the proposed approach effectively generates a well-tuned strategy for white-box RGPO jamming. Overall, the research in this paper lays a foundation for the optimization model and the main framework of algorithm for subsequent related research.

## Figures and Tables

**Table 2:** The average $E[\xi_{\varphi }^{\left(K\right)}]$, $E[{\mathrm{MD}}_{\varphi }^{\left(K\right)}]$, and E[r(φ)] performances of strategies obtained by the proposed method and $E[\xi_{\varphi }^{\left(K\right)}]$, $E[{\mathrm{MD}}_{\varphi }^{\left(K\right)}]$, and E[r(φ)] performances of the best UV-RGPO strategy and UA-RGPO strategy on the CV benchmark with various *K*.

*K*	Method	$E[\xi_{\varphi }^{\left(K\right)}]$	$E[{\mathrm{MD}}_{\varphi }^{\left(K\right)}]$	E[r(φ)]
10	Proposed method	21.4%	140	0.415
10	UV-RGPO	17.7%	113	0.372
10	UA-RGPO	13.3%	126	0.382
15	Proposed method	31.8%	224	0.720
15	UV-RGPO	28.0%	153	0.524
15	UA-RGPO	21.0%	196	0.595
20	Proposed method	37.1%	310	0.920
20	UV-RGPO	35,4%	187	0.645
20	UA-RGPO	20.9%	301	0.856

**Table 3:** The average $E[\xi_{\varphi }^{\left(K\right)}]$, $E[{\mathrm{MD}}_{\varphi }^{\left(K\right)}]$, and E[r(φ)] performances of strategies obtained by the proposed method and $E[\xi_{\varphi }^{\left(K\right)}]$, $E[{\mathrm{MD}}_{\varphi }^{\left(K\right)}]$, and E[r(φ)] performances of the best UV-RGPO strategy and UA-RGPO strategy on the CV benchmark with various $w_{1}$ and $w_{2}$.

$w_{1},w_{2}$	Method	$E[\xi_{\varphi }^{\left(K\right)}]$	$E[{\mathrm{MD}}_{\varphi }^{\left(K\right)}]$	E[r(φ)]
0.25, 0.75/200	Proposed method	30.2%	226	0.920
0.25, 0.75/200	UV-RGPO	27.7%	153	0.642
0.25, 0.75/200	UA-RGPO	19.7%	203	0.811
0.5, 0.5/200	Proposed method	31.8%	224	0.720
0.5, 0.5/200	UV-RGPO	28.0%	153	0.524
0.5, 0.5/200	UA-RGPO	21.0%	196	0.595
0.75, 0.25/200	Proposed method	34.4%	204	0.513
0.75, 0.25/200	UV-RGPO	31.8%	143	0.421
e 0.75, 0.25/200	UA-RGPO	27.4%	173	0.422

**Table 4:** The average $E[\xi_{\varphi }^{\left(K\right)}]$, $E[{\mathrm{MD}}_{\varphi }^{\left(K\right)}]$, and E[r(φ)] performances of strategies obtained by the proposed method and $E[\xi_{\varphi }^{\left(K\right)}]$, $E[{\mathrm{MD}}_{\varphi }^{\left(K\right)}]$, and E[r(φ)] performances of the best UV-RGPO strategy and UA-RGPO strategy on different benchmarks.

Benchmark	Method	$E[\xi_{\varphi }^{\left(K\right)}]$	$E[{\mathrm{MD}}_{\varphi }^{\left(K\right)}]$	E[r(φ)]
CA	Proposed method	49.8%	365	1,16
CA	UV-RGPO	38.9%	223	0.752
CA	UA-RGPO	35.5%	330	1.00
CT	Proposed method	29.7%	208	0.668
CT	UV-RGPO	25.7%	148	0.499
CT	UA-RGPO	18.1%	190	0.568
RV	Proposed method	27.2%	174	0.570
RV	UV-RGPO	26.1%	124	0.441
RV	UA-RGPO	22.3%	148	0.482

## Data Availability

Data are contained within the article. All data in this paper are generated by simulation, and the details have been presented in Section 4.
